# Population expansions dominate demographic histories of endemic and widespread Pacific reef fishes

**DOI:** 10.1038/srep40519

**Published:** 2017-01-16

**Authors:** Erwan Delrieu-Trottin, Stefano Mona, Jeffrey Maynard, Valentina Neglia, Michel Veuille, Serge Planes

**Affiliations:** 1 Laboratoire d’Excellence «CORAIL», EPHE, PSL Research University, UPVD, CNRS, USR 3278 CRIOBE, F-66360 Perpignan, France; 2 Instituto de Ciencias Ambientales y Evolutivas, Universidad Austral de Chile, Valdivia, Chile; 3 Institut de Systématique, Évolution, Biodiversité (ISYEB), UMR 7205 - CNRS, MNHN, UPMC, EPHE, Ecole Pratique des Hautes Etudes, Paris Sorbonne Universités, Paris, France; 4 EPHE, PSL Research University, Paris, France; 5 SymbioSeas and Marine Applied Research Center, Wilmington NC 28411, United States of America

## Abstract

Despite the unique nature of endemic species, their origin and population history remain poorly studied. We investigated the population history of 28 coral reef fish species, close related, from the Gambier and Marquesas Islands, from five families, with range size varying from widespread to small-range endemic. We analyzed both mitochondrial and nuclear sequence data using neutrality test and Bayesian analysis (EBSP and ABC). We found evidence for demographic expansions for most species (24 of 28), irrespective of range size, reproduction strategy or archipelago. The timing of the expansions varied greatly among species, from 8,000 to 2,000,000 years ago. The typical hypothesis for reef fish that links population expansions to the Last Glacial Maximum fit for 14 of the 24 demographic expansions. We propose two evolutionary processes that could lead to expansions older than the LGM: (a) we are retrieving the signature of an old colonization process for widespread, large-range endemic and paleoendemic species or (b) speciation; the expansion reflects the birth of the species for neoendemic species. We show for the first time that the demographic histories of endemic and widespread reef fish are not distinctly different and suggest that a number of processes drive endemism.

The existence of endemic coral reef fish species is a challenge for evolutionary biologists to explain given tropical waters are widely connected. The highest levels of endemism on coral reefs are observed near islands at the peripheries of the Indo-Malay-Philippines Archipelago (IMPA), global hotspot of reef fish species diversity^1^,^2^,^3^,^4^,^5^. As examples from the Pacific Ocean, the percentage of reef fish that are endemic in the Hawaiian archipelago is 25%^6^, 22% in Easter Island^7^, and 14% in the Marquesas Islands^8^. Even if actual patterns of distribution of coral reef fish species are now well depicted, the evolution and processes underlying the establishment and maintenance of endemic species remains unclear. Despite their unique nature and their potentially higher risk of extinction, the origin and population history of endemic species is poorly studied.

The Pleistocene era (c. 1.8–0.01 Ma), which affected the distribution and demographic history of both terrestrial and costal marine species^9^, was characterized by glacial cycles and sea level fluctuations up to 150 m below present sea level^10^,^11^. Large parts of continental shelves were exposed during low sea level, altering shallow water habitat and likely reducing coral reef area^12^,^13^,^14^. These dramatic changes to the environment influence the demographic history of populations, leaving a footprint in the pattern of genetic diversity^15^ that will vary depending on the range extent of the species (i.e., how much of the global species was affected by the event). Population bottlenecks and expansions have often been retrieved in marine populations and shown to coincide with the last major sea level changes impacting population dynamics^9^,^16^,^17^,^18^.

Species biology can affect how reef fish species respond in term of population size variation to major sea level changes such as through varying the larval phase or habitat^19^,^20^,^21^,^22^. However, very few studies have explored how species with different range size respond to major climatic events^23^,^24^. Endemic species have by definition a limited distribution^25^ so are widely expected to be highly vulnerable to environmental changes that are locally disrupting^26^,^27^. This is particularly the case for coral reef fishes forming metapopulations where connectivity occurs only during the larval stage, *i.e.* colonization of new suitable habitats and migrant exchanges among already established populations. Populations of widespread species even at the edges of their geographic range can still be considered as part of a large metapopulation. The exchange of migrants through larval connectivity, even if very infrequent, can enable recovery from major environmental changes^28^. In contrast, endemic species with a limited geographical distribution cannot rely on outcrossing with source populations, being either a newly established species (neoendemism) or the remnant of an ancestral widespread species (paleoendemism). In summary, two main differences characterize endemic *vs* widespread species: the size of the habitat and the degree of connectivity. We therefore expect to see major differences in the demographic histories of endemic and widespread species.

We investigate the genetic diversity and demographic history of 28 reef fish species from the Gambier and Marquesas archipelagos, representing five major reef fish families and selected to include both endemic and widespread species of the same genus. In particular, we examine whether range size is a determinant of the demographic history we retrieve; i.e., do demographic histories vary with range size? Examining multiple pairs of close related species allows us to infer if co-distributed species shared the same demographic history, and test the potential influence of their range distribution. We used both mitochondrial and nuclear DNA sequences to examine demographic history. The use of several independent markers provides a replicate of the coalescent process and improves demographic estimates by reducing the coalescent variance^29^,^30^,^31^. Historically, mitochondrial markers have been widely used in population genetics because of the availability of universal primers (at least for groups of related species) and because of the lack of intralocus recombination, which may bias demographic inferences. However, nuclear markers can now be more easily typed and can also be used to examine demographic history. Using both marker types can enhance our ability to depict complex evolutionary history of species^32^. This study represents the first large-scale comparison of the demographic history of endemic versus widespread marine species. We resolve the demographic histories of the subject species and discuss the potential underlying evolutionary processes that led to present day patterns.

## Results

### Genetic diversity

The 28 species revealed a wide range of genetic variability (Table 1). Of the four factors used as predictors of haplotype and nucleotide diversity for the 4 genes, the only significant predictors were archipelago for nucleotide diversity of the GnRH intron (W = 82, p-value < 0.05; higher values for Gambier Islands) and families for the S7 intron (*H*_*4*_ = 10.2945, p-value < 0.05; with higher values for Serranidae and lower for Chaetodontidae). Haplotype and nucleotide diversity were also not significantly different when analyzed by congener pairs made up of an endemic and widespread species (COI: *h: V* = 21, p = 0.32; π: V = 14, p = 0.34; cytb: *h: V* = 29, p = 0.50; π: V = 11, p = 1; GnRH: *h: V* = 34, p = 0.56; π: V = 21, p = 0.73; S7: *h: V* = 21, p = 0.91; π: V = 19, p = 0.72). Overall haplotype diversity was higher for cytochrome b (0.754 ± 0.197) and S7 intron (0.773 ± 0.231) than for COI (0.598 ± 0.227) and GnRH intron (0.432 ± 0.255). Nucleotide diversity was higher for S7 (0.008 ± 0.007) than for cytochrome b (0.003 ± 0.003), COI (0.002 ± 0.002) or GnRH intron (0.002 ± 0.002).

### Past population size changes

Overall, we retrieved population expansion for almost all species, regardless of their range size or locality. Indeed, we found population expansions for 24 species and a constant effective size for 4 species (Tables 2 and 3).

**Figure 1:**
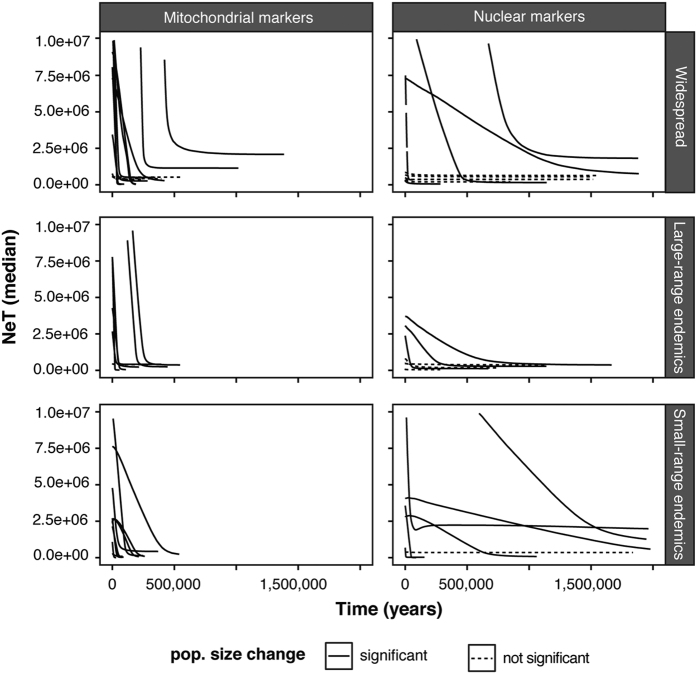
EBSP representing the median of the NeT through time in years for all range size classifications and the two marker types. Line shape denotes species that display a significant expansion (solid line) from species that display no significant change in their population size, i.e. constant population size (dotted line).

Neutrality tests and Extended Bayesian Skyline Plot (EBSP) analysis allowed us to retrieve population expansion for 24 species. For 19 species, we found a population expansion with significant neutrality test *F*_*S*_ for at least one gene for both type of marker, EBSP rejecting a constant size model in favor of one demographic change and a clear graphical expansion (Table 2, Figs 1 and S1). For 5 species, we retrieved a population expansion only from the mitochondrial markers while nuclear markers indicated a signal of constant population. Approximate Bayesian Computation (ABC) analyses were concordant with negative growth rates retrieved, indicating population expansions (Table 2). The species for which expansions were found (19 of 28 with two types of marker, 24 of 28 with one type of marker) are from both archipelagos and include both endemic (5 large-range, 8 small -range) and widespread species (8 species), 4 of the 5 families (except *Chaetodontidae*), and all types of reproduction (Table 2).

We find a constant effective population size only for 4 species (Tables 2 and 3). For these, neutrality tests and EBSP analysis indicated a constant population size for either both type of markers (*Plectroglyphidodon lacrymatus*) or most of the analyses (*Chaetodon declivis, C. citrinellus* and *Plectroglyphidodon leucozonus*) (Tables 2 and 3). All species are from Marquesas; three are widespread species and one is a large-range endemic species. They belong to two different families (Chaetododontidae and Pomacentridae) that differ in their reproduction strategies (pelagic eggs released *vs.* benthic spawners species).

### Timing of the demographic events

We found overall mostly signal of population expansion (24/28 species), with expansion times that varied widely among species, ranging from 8,000 YBP to 2,000,000 YBP, no matter the method used or the genes screened.

**Figure 2:**
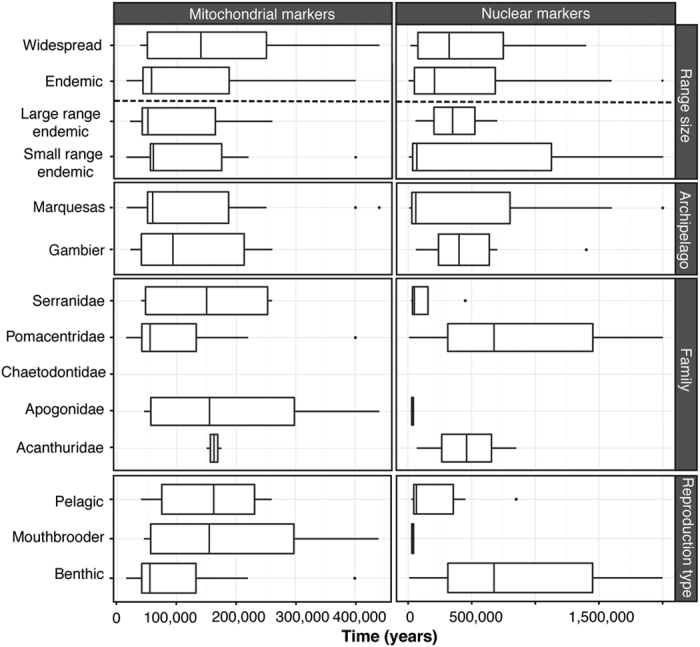
Box and whisker plots of the expansion time (T_EBSP_) for all species and each type of marker, when classified following their range size, the archipelago, their family, and their type of reproduction. Midlines are medians, boxes and whiskers are first/second and third quartiles, respectively, and points are outliers.

For mitochondrial markers, EBSP analysis shows that expansion time varied from 15,000 YBP to 440,000 YBP (Table 3, Fig. 1 and Figs S1 and S2). The timing of expansions retrieved does not differ significantly among species. None of the tested factors were significant: family (*H*_*3*_ = 2.8578, *p* = 0.41), archipelago (W = 55.5, *p* = 0.62), range-size classification (W = 57.5; *p* = 0.57 and *H*_*2*_ = 0.5568, *p* = 0.76), and reproductive strategy (*H*_*2*_ = 2.6737, *p* = 0.26) (Fig. 2 and Fig. S3). For nuclear markers, EBSP analysis shows that expansion times varied more widely among species than was found for mitochondrial markers, ranging from 8,000 YBP to 2,000,000 YBP (Table 3, Figs S1 and S2). As it was the case with the mitochondrial markers, the timing of expansions retrieved for nuclear markers does not differ significantly among species. Again, none of the tested factors were significant: family (*H*_*3*_ = 5.5257, *p* = 0.14), archipelago (W = 38, *p* = 0.43), range-size classification (W = 30, *p* = 1; and *H*_*2*_ = 0.0473, *p* = 0.98), and reproductive strategy (*H*_*2*_ = 4.4632, *p* = 0. 11) (Fig. 2 and Fig. S3).

**Figure 3:**
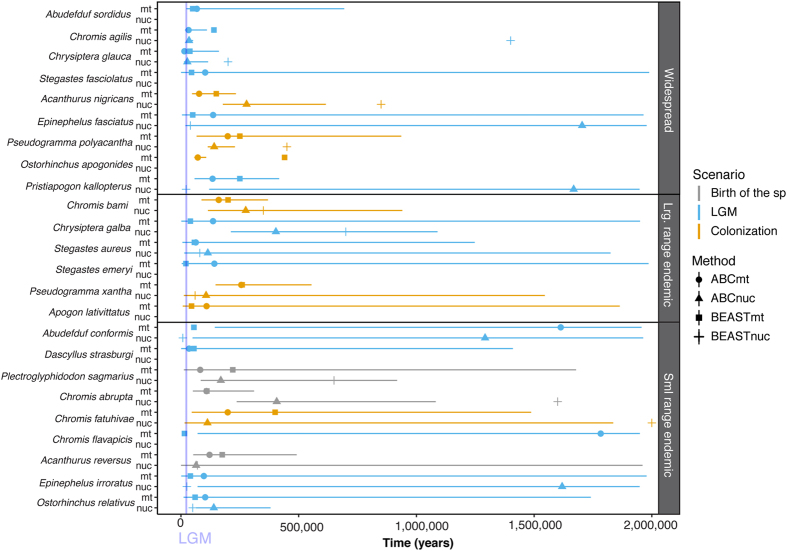
Expansion time retrieved with EBSP and ABC methods. The shape of the dots denotes the method (ABC vs EBSP) applied on each type of marker (mt: mitochondrial; nuc: nuclear). Colors denote potential demographic scenarios associated to the expansions retrieved: Last Glacial Maximum (LGM), colonization of the archipelagos by the species through connectivity (Colonization) or birth of species (Birth of the sp).

We compared the population expansion timing retrieved with the EBSP (T_EBSP_) for the two types of marker. We found a concordant time (within 25,000 years) of expansion for some species (e.g. *Epinephelus irroratus, Ostorhinchus relativus*, or *Stegastes aureus*). For others, T_EBSP_ varied greatly between marker types within the same species (e.g. *Acanthurus nigricans, Pristiapogon kallopterus* or *Chromis fatuhivae*) (Table 3, Fig. 3 and Fig. S2). Considering the 24 species displaying population expansion, we found T_EBSP_ that varied by a factor of 26 for the mitochondrial marker and 250 for the nuclear marker. When narrowed to family, T_EBSP_ varied for Pomacentridae by a factor from 1.7 (Acanthuridae) to 26.7 (Pomacentridae) for the mitochondrial markers and from 2.27 (Apogonidae) to 250 (Pomacentridae) for the nuclear markers. Finally, comparisons between closest relatives (*i.e.* same genus) revealed both very similar T_EBSP_ and very divergent ones, with a variation by a factor of 1.04 (*Pseudogramma*) to 13.33 (*Chromis*) for mitochondrial markers and 1.6 (*Epinephelus*) to 12.14 (*Acanthurus*) for nuclear markers.

Expansion times and credible intervals provided by the ABC analyses (T_ABC_ + CI) relaxed the differences observed for each species considering solely the EBSP expansion time. We retrieved distribution of expansion time (*i.e.* T_EBSP_ with EBSP and T_ABC_ + CI with the ABC) concordant in general but not overlapping for 7 species, mostly due to values retrieved with the EBSP for nuclear marker.

## Discussion

Population expansions dominate the demographic history of endemic and widespread Pacific reef fish. The timing of the population expansions we retrieved varies greatly, between 15,000–440,000 YBP for mitochondrial markers and between 8,000–2,000,000 YBP for nuclear markers (Table 3 and Fig. 3). There are no consistent patterns among the predictor variables with respect to when the expansions likely occurred. Population expansion timing even varied both among and within the endemic and widespread species, which was unexpected. Such expansions have usually been associated with Pleistocene interglacial periods, when sea level variations likely profoundly affected habitat distribution^10^,^33^. Indeed, correlations between population expansion and such Pleistocene sea-level changes have been reported for many reef fish families in the literature: Acanthuridae^19^,^34^,^35^, Chaetodontidae and Pomacentridae^19^, Gobiidae^36^, Holocentridae^37^, Lutjanidae^38^, or Scaridae^39^,^40^, and also for other marine organisms such as gastropods^41^,^42^. Nine glacial cycles have been recorded in the last 800,000 years, all of which resulted in major sea level variations^43^,^44^ that could have produced departures from genetic equilibrium conditions. However, if Pleistocene climate changes drive species demography, we should observe both expansions and population contractions following climatic oscillation. Strikingly, we found two clear patterns only in our survey; (i) population expansion; and (ii) a constant effective size, with the former largely dominating the demographic history of endemic and widespread Pacific reef fish (Table 2). Neither neutrality tests nor EBSP provided any evidences of population contraction for any markers in any species. The statistical power to detect a bottleneck is dependent on the number of markers used^45^. Unfortunately, reconstructing complex demography can be a very difficult task even when analyzing whole genome data (see for example Boitard *et al*.^46^). Using relatively few markers like in this study, it is possible that no coalescence survived the bottleneck preceding the last expansion, erasing all traces of more ancient expansions^47^.

We expected then to recover only the most recent expansion, occurring after the Last Glacial Maximum (LGM), *i.e.*, between 26,500 and 19,000–20,000 years ago^48^. Therefore, we considered that a species expanded because of the climatic change after the LGM if the estimated expansion time was <26,500 years B.P. When looking at T_EBSP_ we found only 5 species for which at least one of the two marker types (*i.e.*, mitochondrial or nuclear) was strictly consistent with an expansion after the LGM (*Abudefduf conformis, Chromis flavapicis, Epinephelus irroratus, Pristiapogon kallopterus*, and *Stegastes emeryi*; Table 3 and Fig. 3). T_EBSP_ is a point estimates inferred visually and a posteriori after the likelihood based analysis performed with BEAST. We therefore performed an ABC estimate of the expansion time to compute its 95% credible interval. We found the distribution of T_ABC_ consistent with the LGM for 9 more species with at least one of the two marker types (*Abudefduf sordidus, Chromis agilis, Chrysiptera galba, C. glauca, Dascyllus strasburgi, Epinephelus fasciatus, Ostorhinchus relativus, Stegastes aureus, S. fasciolatus*; Table 3 and Fig. 3). These 14 species (5 detected with T_EBSP_ and 9 with T_ABC_) probably experienced variations of their population size during the Pleistocene interglacial periods and started expanding soon after their habitats were restored.

However, we retrieved expansion times for both endemic and widespread species older than the LGM for almost half of the species. These results are totally in agreement with previous phylogeographic studies based on mitochondrial markers. Population expansions have been found for reef fish in Marquesas Islands of 15,400–23,900 YBP for *Chaetodon ornatissimus*^49^; 180,000–370,000 YBP for *Lutjanus kasmira* and 120,000–240,000 YBP for *L. fulvus*^38^. We propose two other scenarios as potential explanations of the large range we find in the timing of population expansions and the lack of differences between endemic and widespread species.

- Signals of population expansion observed before the LGM correspond to the colonization of the archipelagos by the species through connectivity. This scenario seems to be more dominant as most expansions were older than the LGM (14/24 expansions retrieved considering expansion and mixed categories (Table 3)). This may apply to widespread species like *Chromis agilis* (mtDNA: 140,000 YBP, nucDNA: 1,400,000 YBP), or *Ostorhinchus apogonoides* (mtDNA: 440,000 YBP) and large-range endemic species like *C. bami* (mtDNA: 200,000 YBP, nucDNA: 350,000 YBP). For these species, migrants coming from other demes of the metapopulation may have recolonized new empty habitat. This scenario may also apply to the small-range endemic species *Chromis fatuhivae* (mtDNA: 400,000 YBP, nucDNA: 2,000,000 YBP), a species presenting an important genetic divergence (11% with cytochrome b, roughly 11Mya with a 1% divergence rate) with its only close relative known to date, *C. bami*^50^. Small-range endemic species can be paleoendemic, i.e. these are the remnants of species that once had a much larger range size.
- The expansions correspond to the actual success of local speciation. This scenario is only likely for relatively young endemic species (i.e. neoendemics). Several endemic species from these regions have demonstrated a young evolutionary history and show incomplete lineage sorting when distance trees are computed with their closest sister species (e.g. *Canthigaster criobe*^51^, *Kuhlia petiti*^52^, *Mulloidichthys mimicus*^53^, or *Acanthurus reversus*^54^). Divergence time constitutes the upper bound of the speciation process and the initiation of the differentiation of species. This scenario may apply to the Marquesan endemic species *Acanthurus reversus* (mtDNA: 175,000 YBP, nucDNA: 70,000 YBP)*, Plectroglyphydodon sagmarius* (mtDNA: 220,000 YBP, nucDNA: 650,000 YBP), and *Chromis abrupta* (mtDNA: 110,000 YBP, nucDNA: 1,600,000 YBP). All of them are the sibling species of widespread species that they replace in Marquesas (respectively *A. olivaceus*^55^, *P. imparipennis* and *C. margaritifer*^50^) and present a young evolutionary history. *A. reversus* shows incomplete lineage sorting with *A. olivaceus*^54^ and so does *C. abrupta* and *C. margaritifer* (many *C. abupta* COI haplotypes matches (100% means identity) *C. margaritifer* COI haplotypes (GenBank numbers: FJ583159; FJ583158; and FJ583162)) while *P. sagmarius* present little genetic divergence with its sibling species, less than 2% (*P. imparipennis* COI sequence number JQ350225 (GenBank)), equivalent to a divergence date of less than 2 Mya. The expansion retrieved for these species would then correspond to their birth as endemic species.

All our results are based on the hypothesis that we correctly specified the mutation rate for all our species and all the genes here considered. Rates may vary greatly from one species to another and the estimates obtained rely heavily on the statistical methods used to calibrate the clock^56^,^57^. Nonetheless, molecular clock rates for COI and cytochrome b for reef fish are well known and we used the 1–2% divergence rate generally used as a consensus in the literature^23^,^49^,^56^,^58^,^59^. Concerning nuclear markers, we used a divergence rate for S7 calibrated on reef fishes^60^ and polar fishes^20^. They constitute the only calibration so far obtained for this intron while no calibration has been proposed yet for the GnRH intron. However, evolution rates for nuclear DNA are usually slower than those for mitochondrial DNA^57^,^61^ and such rate seems to be in accordance with rates of mitochondrial markers we used, though those rates can vary greatly in vertebrates^56^,^62^,^63^. We acknowledge that variability in the molecular rate of evolution among our species may exist and inflate the differences we have observed in the estimated expansion times. Nevertheless, if we consider T_EBSP_, we note that it varies between the youngest and the oldest estimate by a factor of 26 for the mitochondrial markers and 250 for the nuclear markers. Such wide distribution was confirmed when looking at closely related species: expansion times varied for example for Pomacentridae by a factor of 19 for the mitochondrial markers and 200 for the nuclear markers and for *Chromis* genus by a factor of 13,3 for the mitochondrial markers and 5,7 for the nuclear markers. Similar values are found when considering the mode of T_ABC_. In summary, the width of the distribution of those estimated expansion time (both T_EBSP_ and T_ABC_) for both mitochondrial and nuclear markers is so large that it cannot be explained by the uncertainty of the molecular rate or the variance associated with the estimation process. In summary, we cannot reconcile all the dates to a single environmental change. It is much more likely that each species (or at least several groups of species) was affected differently by successive specific events.

The use of two types of molecular markers allowed us to reconstruct the demographic history of reef fishes from two remote archipelagos hosting high level of endemism. We find that the demographic history of most Pacific reef fish species, despite their variety of range size and life traits, is dominated by population expansions. Consistently with the absence of difference in the demographic signature, we find that the genetic diversity of endemic and widespread species is similar. This matches the conclusions of our previous study, which was only based on one mitochondrial marker (cytochrome b)^64^, but see also Eble, *et al*.^23^ and Hobbs, *et al*.^65^. We strengthen this result here by adding another mitochondrial locus and two unlinked nuclear loci. Genetic diversity and signature of effective population size change are therefore not correlated with the range-size distribution of reef fish species. More loci will be needed to refine the demographic trajectories of each species, both to describe events that could have been missed due to a lack of statistical power and to improve the parameter estimation process. Overall, this study highlights that demographic histories of endemic species do not differ from widespread species; both have complex and similar histories.

## Methods

### Specimen collection

**Figure 4:**
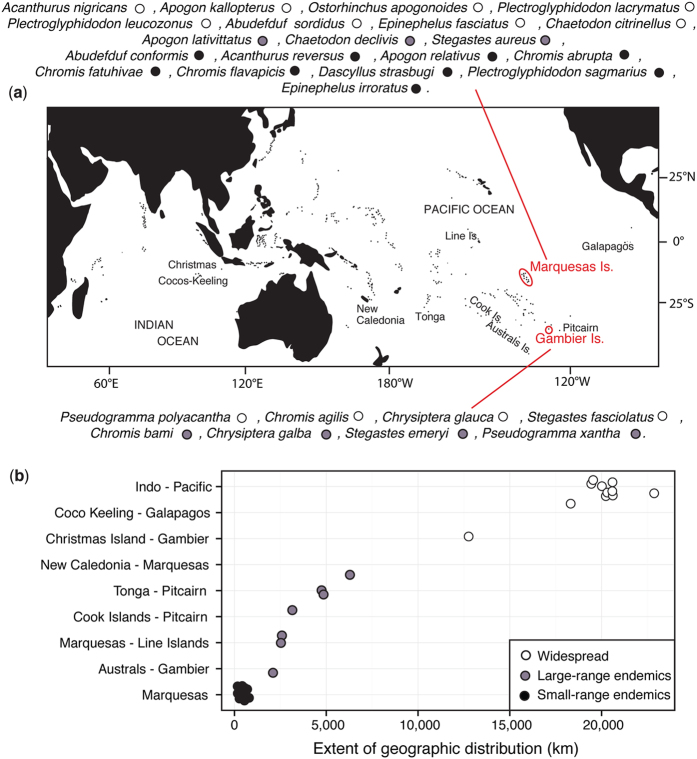
Sampling locations and geographic distributions of the study species. (**a**) Species sampled in Marquesas (20) and Gambier Islands (8) (~45 individuals per species) with their range size. (**b**) The maximum extent of each species is reported with the location itself (e.g. Marquesas) or the westward and eastward know location to date (e.g. Cocos Keeling–Galapagos). The map was produce using R^82^, R package ‘maps’^90^ and modified using Adobe Illustrator CS5 v 15.0.2. http://www.adobe.com/products/illustrator.html.

The Gambier archipelago (centered on 23°S, 134°W) is southeast of the Tuamotu Archipelago; it includes 11 high islands spreading only over 40 km and enclosed by a wide barrier reef. The Marquesas archipelago spread over 500 km between 8°–11°S and 141°–138°W and includes 12 high volcanic islands surrounded by fringing reefs^66^; these are the northeastern-most islands of French Polynesia. We sampled a total of 1,244 reef fishes using polespears or anesthetic while SCUBA diving and exploring all islands of the Gambier Islands and of the Marquesas Islands respectively in 2010 and 2011. This dataset is composed of 28 species (~45 individuals per species), with 20 and 8 co-distributed species respectively in Marquesas and in Gambier Islands (Fig. 4a), and includes a wide range of life history traits (Table 1). Species range distribution (Fig. 4b) varied from widespread (range size > 12 000 km, 13 species), to large-range endemic (1000–8000 km, 6 species) and small-range endemic (less than 500 km, 9 species, see justification in Delrieu-Trottin *et al*.^64^). Five different families are represented: Acanthuridae, Apogonidae, Chaetodontidae, Pomacentridae and Serranidae. Several reproductive strategies are represented meaning larval dispersion potential varies greatly (Table 1). Difference of sampling between the archipelagos relates to the uniqueness of Marquesas Islands. Third highest region of endemism for coral reef fishes in the Indo-Pacific^8^, they host many more cases of endemism.

### Ethics Statement

The study protocol was approved by the National Center for Scientific Research and is in accordance to the laws of the French Republic and of the collectivity of French Polynesia.

### Laboratory procedures and genetic analyses

We extracted whole genomic DNA from fin tissues preserved in 96% EtOH at ambient temperature using QIAxtractor (QIAGEN, Crawley) according to manufacturer’s protocols. Fragments of the cytochrome b for 1,237 individuals (Cyt b, 739–999 bp) and cytochrome C oxydase subunit 1 for 1,005 individuals (COI, 571–688 bp), both parts of mitochondrial genome, were amplified using PCR. These were sequenced using the universal primers GLUDGL-CB3H of Palumbi *et al*.^67^ and different combinations of primers from Ward *et al*.^68^. In addition, part of the third intron in the Gonadotropin-Releasing Hormone gene for 912 individuals (GnRH, 267–417 bp) and part of the first intron of the S7 ribosomal protein gene for 973 individuals (S7, 485–783 bp) were amplified. For these, we used primers GnRH3.3F-GnRH3.3R^69^ and primers S7RPEX1F-S7RPEX2R^70^. Fragments were amplified using PCR protocols and sequencing as described by Williams *et al*.^51^. Differences in the number of samples are due to our inability to amplify cytochrome b for 3 species (*Epinephelus irroratus, Epinephelus fasciatus* and *Ostorhinchus relativus*), GnRH intron for 7 species (*Dascyllus strasburgi, Chromis flavapicis, Chromis abrupta, Apogon lativittatus, Ostorhinchus apogonoides, Ostorhinchus relativus* and *Pristiapogon kallopterus*) and S7 intron for 3 species (*Apogon lativittatus, Epinephelus fasciatus and Ostorhinchus apogonoides*). Overall, we worked with a dataset comprised of at least one mitochondrial marker and one nuclear marker for 26 species. Four markers were available for 19 species, 3 markers for 5 species and 2 markers for 4 species. Only mitochondrial data were available for 2 species (Table 1).

Sequences were edited using GENEIOUS PRO v.6.1.7 (Biomatters) and aligned with Clustal W^71^ as implemented in GENEIOUS. Alignments were unambiguous with no indels or frameshift mutations. For the nuclear markers, allelic state from sequences with multiple heterozygous single nucleotide polymorphisms (SNPs) was estimated using PHASE v.2.1.1 as implemented in DnaSP^72^,^73^,^74^,^75^. We tested for recombination using SBP and GARD methods implemented online on the Datamonkey webserver^76^,^77^. The GARD and SBP tests failed to find recombination for any of the nuclear markers of either species.

Haplotype diversity (*h*) and nucleotide diversity (π) were calculated using the software package DnaSP v.5.1^75^. To detect departures from a neutral Wright–Fisher model, we used Fu’s *F*_*S*_ neutrality test^78^. Assuming selective neutrality, significant negative values of *F*_*S*_ indicate population growth while significant positive values are a signature of either genetic subdivision or population contraction. The test was implemented in DnaSP v.5.1^77^ and its significance was determined from 1000 coalescent simulations.

Changes in effective population size (Ne) through time were estimated using the Extended Bayesian Skyline Plot (EBSP) implemented in BEAST v.1.7^79^,^80^. EBSP is a non-parametric model that does not specify any prior hypothesis on the tempo and mode of changes of the Ne. EBSP allows the analysis of multiple loci, reducing the stochastic variance of the coalescent process and improving the reliability of demographic inferences^47^. Mitochondrial and nuclear markers have different mechanisms of evolution and inheritance, which are also affected by reproductive strategies and sex-biased processes. The species investigated use a range of reproductive strategies (e.g. sequential hermaphrodites *vs* gonochoristic species, couple *vs* harem) so we analyze mtDNA and nuclear markers separately in the EBSP analyses.

The two nuclear loci are unlinked so have different genealogies, substitution models and clock rates but the same underlying demography. As the mitochondrial genes are in linkage by structure, we used different substitution models and clock rates but the same genealogy. Sequence divergence estimates for the two mitochondrial markers (COI and Cyt b) in reef fish range from 1% to 2% per million years^56^,^58^,^59^,^81^. We set a strict clock with a uniform prior distribution for the clock rate with an upper and lower mutation rate ranging from 0.5 × 10^−8^ to 1 × 10^−8^ per site per year. We retrieved various sequence divergence estimates for S7 intron from 0.28% to 1.7%^20^,^56^,^60^ while no divergence rate was available in the literature for the third intron in the Gonadotropin-Releasing Hormone (GnRH). For these two introns, we set a strict clock with a uniform prior distribution for the clock rate with an upper and lower mutation rate ranging from 0.14 × 10^−8^ to 0.85 × 10^−8^ per site per year. To take into account possible site-specific variation in the mutation rate we used the HKY + G model of mutation for all genes. We ran 10 million MCMC iterations with a thinning interval of 1,000. We checked convergence by visually inspecting the trace and computing the effective sample size (which was always higher than 100) for each parameter in two independent runs using the program TRACER v.1.5 (http://tree.bio.ed.ac.uk/software/tracer/). EBSP were plotted using R v.3.2.3^82^ (R Development Core Team 2015) setting the burn-in to 10%.

Generation time is unknown for most species selected for this study and it potentially varies greatly among them as we selected a large range of families. To remove that uncertainty, we therefore set the mutation rate in units per site per year to obtain time points expressed in calendar years; independent of the generation time^80^. That method allows comparing potential expansion times between close related species but also different families. The effective size being scaled by the generation time (unknown for most species), only the timing of expansions between species will be discussed in the present work.

Finally, we compute the posterior distribution of the number of demographic changes occurring along the gene genealogies. This method formally tests how many times Ne changed through the history of the gene genealogy, allowing to reject a constant size population model. We rejected a constant population size model if the lower bound of the 95% high posterior density (HPD) of this distribution was higher than zero. Expansion times were identified from the skyline output as the point when the Ne started to increase.

The data did not meet assumptions of normality so we computed the non-parametric tests Wilcoxon-Mann-Whitney and Kruskal-Wallis to examine differences in genetic diversity (haplotype and nucleotide) and demography (TMRCA, time of expansion) among four factors: range size (endemic vs. widespread and all three range types), archipelagos, families, and reproductive strategies. We also computed Wilcoxon signed-rank test to compare the genetic diversity indices for endemic species that had a widespread congener (*i.e.* same genus) caught in the same archipelago. All statistical analyses were performed on R, using the vegan package^83^, and ggplot2 for graphics^84^.

To compute the full posterior distribution of the expansion time (T) for each species we developed an Approximate Bayesian Computation (ABC) approach^85^. ABC summarizes the probability of the data through a vector of observed summary statistics and it is therefore very flexible as it can investigate any demographic model provided it can be simulated^86^. EBSP showed that all species were either expanding (following approximately an exponential growth) or constant (see results). EBSP uses a likelihood function to compute the probability of the observed data and it is therefore to be preferred over methods which approximate the likelihood. However, the EBSP does not compute explicitly the T_exp_, which is inferred visually a posteriori and has thus no credible interval associated. We therefore implemented the following three parameters model with ABC: an ancestral constant population characterized by an effective population size (N_anc_) starts to increase (or decrease) exponentially at T_exp_ to reach at time 0 the modern effective population size (N_mod_). We assigned the following uniform priors to the three parameters: N_anc_ and N_mod_: {1,000 ÷ 10,000,000}; T_exp_: {100 ÷ 2,000,000}. Mutation rate was set in years as in the EBSP; we used a value of 10^−8^ per site per year for the mtDNA genes and 8 * 10^−9^ for the nuclear genes. We performed 500,000 coalescent simulations with parameter values extracted from prior distributions using fastsimcoal2 v.2.5.1^87^. We computed as summary statistics the number of haplotype, the number of segregating sites, the mean pairwise difference and Fu’s Fs using alrsumstat^88^. Parameters were estimated from the 5,000 simulations closest to the observed dataset using a local linear regression according to Beaumont *et al*.^85^ as implemented under the R environment in the library *abc*^89^. Analyses were performed separately for the whole mtDNA (one genealogy) and the two nuclear genes (two independent genealogies with the same underlying demography) as in the EBSP.

## Additional Information

**Accession codes:** Sequences have been deposited in GenBank; GenBank accession number KM455125–KM455538 for cytochrome b (unique sequences); KY207629–KY207951 for COI (unique sequences); KY207952–KY208907 for GnRH intron and KY208908–KY209890 for S7 intron.

**How to cite this article:** Delrieu-Trottin, E. *et al*. Population expansions dominate demographic histories of endemic and widespread Pacific reef fishes. *Sci. Rep.* **7**, 40519; doi: 10.1038/srep40519 (2017).

**Publisher's note:** Springer Nature remains neutral with regard to jurisdictional claims in published maps and institutional affiliations.

## Supplementary Material

Supplementary Information

## Figures and Tables

**Table 1:** Genetic diversity for all species.

Range size	Family	Species (nb of ind.)	Loc	Cyt b		COI		GnRH		S7	
				*h*	π	*h*	π	*h*	π	*h*	π
W	Acanthuridae [p]	*Acanthurus nigricans* (35, 33, 38, 24)	M	0.881	0.002	0.799	0.002	0.586	0.002	0.996	0.014
W	Apogonidae [m]	*Pristiapogon kallopterus* (35, 43, −, 40)	M	0.965	0.006	0.687	0.002	—	—	0.330	0.002
W	Apogonidae [m]	*Ostorhinchus apogonoides* (42, 42, −, −)	M	0.981	0.006	0.834	0.004	—	—	—	—
W	Chaetodontidae [p]	*Chaetodon citrinellus* (45, 48, 48, 46)	M	0.784	0.002	0.590	0.001	0.101	0.0003	0.829	0.006
W	Pomacentridae [b]	*Abudefduf sordidus* (48, 50, 50, 48)	M	0.824	0.002	0.749	0.003	0.686	0.002	0.941	0.010
W	Pomacentridae [b]	*Chromis agilis* (47, 50, 50, 47)	G	0.902	0.003	0.659	0.002	0.902	0.003	0.980	0.015
W	Pomacentridae [b]	*Chrysiptera glauca* (50, 51, 50, 50)	G	0.690	0.001	0.291	0.001	0.690	0.001	0.686	0.004
W	Serranidae [p]	*Epinephelus fasciatus* (−, 50, 45, −)	M	—	—	0.740	0.002	0.262	0.001	—	—
W	Pomacentridae [b]	*Plectroglyphidodon lacrymatus* (43, 48, 48, 20)	M	0.854	0.003	0.724	0.003	0.061	0.0002	0.726	0.011
W	Pomacentridae [b]	*Plectroglyphidodon leucozonus* (46, 46, 46, 46)	M	0.554	0.004	0.554	0.001	0.293	0.001	0.571	0.005
W	Pomacentridae [b]	*Stegastes fasciolatus* (25, 35, 39, 21)	G	0.490	0.001	0.588	0.001	0.490	0.001	0.884	0.008
W	Serranidae [p]	*Pseudogramma polyacantha* (44, 38, 43, 33)	G	0.987	0.011	0.969	0.007	0.987	0.011	0.986	0.009
L	Apogonidae [m]	*Apogon lativittatus* (50, 44, −, 38)	M	0.819	0.004	0.553	0.002	—	—	—	—
L	Chaetodontidae [p]	*Chaetodon declivis* (45, 43, 44, 37)	M	0.771	0.004	0.445	0.001	0.250	0.001	0.880	0.008
L	Pomacentridae [b]	*Chromis bami* (42, 41, 46, 42)	G	0.931	0.004	0.949	0.005	0.931	0.004	0.911	0.008
L	Pomacentridae [b]	*Chrysiptera galba* (44, 45, 45, 45)	G	0.741	0.002	0.445	0.001	0.741	0.002	0.953	0.011
L	Pomacentridae [b]	*Stegastes aureus* (41, 34, 41, 41)	M	0.622	0.001	0.520	0.001	0.806	0.004	0.907	0.003
L	Pomacentridae [b]	*Stegastes emeryi* (48, 49, 48, 47)	G	0.659	0.001	0.158	0.0003	0.659	0.001	0.584	0.001
L	Serranidae [p]	*Pseudogramma xantha* (22, 22, 21, 22)	G	0.996	0.008	0.714	0.003	0.996	0.008	0.758	0.002
S	Pomacentridae [b]	*Abudefduf conformis* (35, 42, 43, 37)	M	0.301	0.0004	0.376	0.001	0.134	0.0004	0.176	0.0003
S	Acanthuridae [p]	*Acanthurus reversus* (48, 47, 48, 47)	M	0.828	0.002	0.855	0.003	0.513	0.002	0.957	0.026
S	Apogonidae [m]	*Ostorhinchus relativus* (−, 48, −, 38)	M	—	—	0.658	0.002	—	—	0.358	0.001
S	Pomacentridae [b]	*Chromis abrupta* (49, 45, −, 44)	M	0.677	0.002	0.525	0.001	—	—	0.973	0.013
S	Pomacentridae [b]	*Chromis fatuhivae* (30, 35, 35, 27)	M	0.959	0.007	0.931	0.005	0.376	0.002	0.971	0.020
S	Pomacentridae [b]	*Chromis flavapicis* (44, 45, −, 24)	M	0.285	0.0004	0.088	0.0001	—	—	0.550	0.006
S	Pomacentridae [b]	*Dascyllus strasbugi* (45, 45, −, 42)	M	0.595	0.001	0.463	0.001	—	—	0.960	0.018
S	Serranidae [p]	*Epinephelus irroratus* (−, 41, 39, 38)	M	—	—	0.352	0.0001	0.146	0.0004	0.684	0.002
S	Pomacentridae [b]	*Plectroglyphidodon sagmarius* (44, 41, 45, 29)	M	0.933	0.004	0.773	0.002	0.323	0.002	0.978	0.010

Species names, sampling locations (G, Gambier archipelago; M, Marquesas Islands) and associated diversity indices (*h:* haplotype diversity; π, nucleotide diversity), structured by range-size classification (W: Widespread species; L: large-range endemic species: S: small-range endemic species). Codes for reproductive strategy are: [p] eggs released in pelagic environment, [b] eggs laid on the bottom and [m] mouthbrooding.

**Table 2:** Summary of the neutrality tests (Fu’ Fs), EBSP analysis and ABC analysis for the 28 species.

Species	Mitochondrial markers						Nuclear markers					
			EBSP pop. size change	ABC					EBSP pop. size change	ABC		
	*F*_*S*_			Growth rate			*F*_*S*_			Growth rate		
	Cytb	COI		Mode	95% low	95% up	GnRH	S7		Mode	95% low	95% up
*A. nigricans*^*W*^	**−19.30**	**−5.13**	Exp*	**−**0,000066	**−**0,000092	**−**0,000022	**−**2.71	**−34.05**	Exp*	**−**0,000009	**−**0,000034	0,000001
*P. kallopterus*^*W*^	**−18.53**	**−11.36**	Exp*	**−**0,000027	**−**0,000063	**−**0,000007	—	**−**3.97	Exp*	0,000037	0,000036	0,000037
*O. apogonoides*^*W*^	**−31.03**	**−14.67**	Exp*	**−**0,000047	**−**0,000056	**−**0,000043	—	—	—	—	—	—
*C. citrinellus*^*W*^	**−**3.14	**−**1.15	ns-cst	0,000002	**−**0,000051	0,000009	**−4.28**	**−**1.52	ns-cst	0,000002	**−**0,000191	0,000007
*A. sordidus*^*W*^	**−21.74**	**−**1.85	Exp*	**−**0,000037	**−**0,000098	0,000013	**−**1.25	**−7.03**	ns-cst	**−**0,000021	**−**0,000230	**−**0,000014
*C. agilis*^*W*^	**−16.12**	**−10.23**	Exp*	**−**0,000055	**−**0,000094	**−**0,000004	**−8.91**	**−37.07**	Exp*	0,000001	**−**0,000110	0,000020
*C.glauca*^*W*^	**−21.00**	**−7.63**	Exp*	**−**0,000099	**−**0,000100	0,000007	**−12.16**	**−15.34**	Exp*	0,000045	**−**0,000647	0,000111
*E. fasciatus*^*W*^	—	**−5.35**	Exp*	0,000007	**−**0,000100	0,000043	**−3.29**	**—**	Exp*	0,000004	**−**0,000138	0,000009
*P. lacrymatus*^*W*^	**−**1.92	**−**2.42	ns-cst	0,000005	**−**0,000023	0,000008	**−**1.01	2.51	ns-cst	0,000012	**−**0,000017	0,000015
*P. leucozonus*^*W*^	2.24	**−3.44**	ns-cst	0,000003	**−**0,000015	0,000009	**−**1.57	**−**1.19	ns-cst	0,000001	**−**0,000056	0,000006
*S. fasciolatus*^*W*^	**−5.97**	**−**0.41	Exp*	**−**0,000094	**−**0,000100	0,000094	**−4.32**	**−**2.18	ns-cst	**−**0,000002	**−**0,000103	0,000001
*P. polyacantha*^*W*^	**−25.33**	**−17.82**	Exp*	**−**0,000012	**−**0,000030	**−**0,000006	**−3.37**	**−68.84**	Exp*	0,000130	**−**0,000847	0,000566
*A. lativittatus*^*L*^	**−9.72**	**−3.40**	Exp*				—	—	—	—	—	—
*C. declivis*^*L*^	**−**0.21	0.11	ns-cst	0,000002	**−**0,000013	0,000012	**−3.45**	**−**3.24	ns-cst	0,000007	**−**0,000417	0,000012
*C. bami*^*L*^	**−5.83**	**−22.85**	Exp*	**−**0,000027	**−**0,000070	**−**0,000009	**−11.06**	**−20.26**	Exp*	**−**0,000015	**−**0,000043	**−**0,000009
*C. galba*^*L*^	**−20.09**	**−**1.62	Exp*	0,000031	**−**0,000100	0,000086	**−29.64**	**−20.63**	Exp*	**−**0,000006	**−**0,000016	**−**0,000004
*S. aureus*^*L*^	**−8.26**	**−2.79**	Exp*	**−**0,000049	**−**0,000100	0,000014	**−**2.77	**−10.75**	Exp*	0,000012	**−**0,000047	0,000015
*S. emeryi*^*L*^	**−5.21**	**−5.51**	Exp*	**−**0,000094	**−**0,000100	0,000095	**−**0.55	0.09	ns-cst	0,000001	**−**0,000033	0,000017
*P. xantha*^*L*^	**−15.86**	**−7.60**	Exp*	**−**0,000013	**−**0,000024	**−**0,000007	**−**1.77	**−7.63**	Exp*	**−**0,000017	**−**0,000239	**−**0,000003
*A. conformis*^*S*^	**−**0.76	**−2.75**	Exp*	0,000002	**−**0,000013	0,000030	**−3.62**	**−**1.19	Exp*	0,000005	**−**0,000189	0,000029
*A. reversus*^*S*^	**−11.30**	**−8.08**	Exp*	**−**0,000023	**−**0,000054	**−**0,000006	**−4.50**	**−**5.38	Exp*	0,000003	**−**0,000251	0,000008
*O. relativus*^*S*^	—	**−9.26**	Exp*	**−**0,000022	**−**0,000100	0,000011	—	**−20.73**	Exp*	**−**0,000025	**−**0,000071	**−**0,000016
*C. abrupta*^*S*^	**−17.88**	**−9.75**	Exp*	**−**0,000043	**−**0,000091	**−**0,000016	—	**−31.38**	Exp*	**−**0,000010	**−**0,000020	**−**0,000004
*C. fatuhivae*^*S*^	**−12.70**	**−11.93**	Exp*	**−**0,000013	**−**0,000043	**−**0,000001	**−**2.10	**−13.60**	Exp*	**−**0,000003	**−**0,000031	0,000000
*C. flavapicis*^*S*^	**−**2.13	**−2.89**	Exp*	0,000003	**−**0,000099	0,000038	—	0.75	ns-cst	0,000007	**−**0,000010	0,000008
*D. strasbugi*^*S*^	**−3.80**	**−9.92**	Exp*	**−**0,000095	**−**0,000100	0,000100	—	**−**5.58	ns-cst	0,000011	0,000000	0,000012
*E. irroratus*^*S*^	**—**	**−6.22**	Exp*	**−**0,000094	**−**0,000100	0,000085	**−**1.73	**−5.4**	Exp*	0,000003	**−**0,000036	0,000015
*P. sagmarius*^*S*^	**−10.12**	**−8.37**	Exp*	**−**0,000019	**−**0,000100	0,000017	**−7.28**	**−20.23**	Exp*	**−**0,000008	**−**0,000043	**−**0,000001

Neutrality tests are computed per gene (significant test results are in bold) while demographic change tests (EBSP) are computed for each type of marker (mitochondrial and nuclear). Significance (ie. rejection or not of a constant population size model) is reported with an asterisk (*) and the trend of the curve of Ne through time is then reported (Exp: expansion; cst: constant). The mode and the credible interval (9(% low- 95% up) of the growth rate retrieved with the ABC analysis are reported for each type of marker. Superscripts next to species names refer to range size classifications (W for widespread, L for large-range endemic, and S for small-range endemic).

**Table 3:** Summary of the demographic pattern and potential demographic scenarios retrieved for the 28 species.

Loc		Species	Demographic pattern	Mitochondrial markers		Nuclear markers		Scenario
				EBSP mt	ABC mode (95% low–95% up)	EBSP nc	ABC mode (95% low–95% up)	
Marquesas	Acanthuridae	*A. nigricans*^*W*^	Expansion	150,000	77,150 (47,327–233,321)	850,000	278,741 (178,005–615,493)	Colonization
		*A. reversus*^*S*^	Expansion	1,750,000	121,515 (52,479–491,637)	70,000	64,428 (523–1,960,596)	Birth of the sp
	Apogonidae	*P. kallopterus*^*W*^	Expansion	250,000	134,490 (57,628–416,920)	22,000	1,667,951 (120,090–1,947,919)	LGM
		*O. apogonoides*^*W*^	Expansion	440,000	71,611 (56,881–107,037)	NA		Colonization
		*A. lativittatus*^*L*^	Expansion	45,000		NA	NA	Colonization
		*O. relativus*^*S*^	Expansion	60,000	103,100 (11,136–1,740,596)	50,000	139,451 (64,397–380,333)	LGM
	Chaetodontidae	*C. citrinellus*^*W*^	Constant	cst	—	cst	—	cst
		*C. declivis*^*L*^	Constant	cst	—	cst	—	cst
	Pomacentridae	*A. sordidus*^*W*^	Expansion	50,000	67,678 (22,769–693,539)	NA	NA	LGM
		*P. lacrymatus*^*W*^	Constant	cst	—	cst	—	cst
		*P. leucozonus*^*W*^	Constant	cst	—	cst	—	cst
		*S. aureus*^*L*^	Expansion	57,000	63,157 (6,195–1,247,667)	80,000	114,031 (13,954–1,824,675)	LGM
		*A. conformis*^*S*^	Expansion	55,000	1,613,112 (143,757–1,956,271)	8,000	1,291,656 (49,136–1,963,343)	LGM
		*C. abrupta*^*S*^	Expansion	110,000	108,634 (50,681–310,156)	1,600,000	406,701 (236,840–1,082,108)	Birth of the sp
		*C. fatuhivae*^*S*^	Expansion	400,000	198,602 (45,884–1,487,344)	2,000,000	112,534 (15,241–1,835,716)	Colonization
		*C. flavapicis*^*S*^	Expansion	15,000	1,782,906 (70487–1,949,050)	cst	—	LGM
		*D. strasbugi*^*S*^	Expansion	55,000	34,411 (876–1,409,771)	cst	—	LGM
		*P. sagmarius*^*S*^	Expansion	220,000	81,947 (12,372–1,678,216)	650,000	169,329 (84,110–917,985)	Birth of the sp
Serranidae	*E. fasciatus*^*W*^	Expansion	50,000	136,583 (4,483–1,964,767)	40,000	1,703,784 (18,905–1,977,880)	LGM	
	*E. irroratus*^*S*^	Expansion	40,000	97,261 (1,299–1,977,553)	25,000	1,618,898 (70,671 1,948,224)	LGM	
Gambier	Pomacentridae	*Chro. agilis*^*W*^	Expansion	140,000	32,626 (17,134–110,291)	1,400,000	34,253 (28,345–51,460)	LGM
		*Chry. glauca*^*W*^	Expansion	38,000	14,858 (5,598–161,196)	200,000	27,005 (13,083–115,193)	LGM
		*S. fasciolatus*^*W*^	Expansion	45,000	102,871 (547–1,988,674)	NA	NA	LGM
		*Chro. bami*^*L*^	Expansion	200,000	160,420 (87,364–369,412)	350,000	275,113 (114,596–940,228)	Colonization
		*Chry. galba*^*L*^	Expansion	40,000	136,301 (1,498–1,949,759)	700,000	403,177 (211,888–1,089,740)	LGM
		*S. emeryi*^*L*^	Expansion	21,000	142,046 (3,126–1,985,907)	cst	—	LGM
	Serranidae	*P. polyacantha*^*W*^	Expansion	250,000	198,674 (66,498–935,574)	450,000	141,057 (113,689–229,073)	Colonization
		*P. xanthum*^*L*^	Expansion	260,000	256,392 (146,927–554,513)	60,000	106,692 (12,082–1,545,452)	Colonization

Expansion times retrieved from EBSP analysis and the ABC analysis (mode + Credible Interval) are reported when a population expansion (Expansion) is retrieved. Potential demographic scenarios associated with the expansions retrieved: to the Last Glacial Maximum (LGM), to the colonization of the archipelagos by the species through connectivity (Colonization) or to the birth of species (Birth of the sp). Species names, sampling locations (Gambier, Gambier archipelago; Marquesas, Marquesas Islands) structured by family and range-size classification. (W for widespread, L for large-range endemic, and S for small-range endemic).
