# Supplementary material for: Population expansions dominate demographic histories of endemic and widespread Pacific reef fishes
Source: Sci Rep. 2017 Jan 16;7:40519. doi: 10.1038/srep40519 (PMC5238389; doi:10.1038/srep40519)
Supplement: Supplementary Information [file srep40519-s1.doc]

**Supporting information**

**Population expansions dominate demographic histories of endemic and widespread Pacific reef fishes**

Erwan Delrieu-Trottin^1, 2*^, Stefano Mona^3,4^, Jeffrey Maynard^1, 5^ Valentina Neglia^1,2^, Michel Veuille^3,4^, Serge Planes^1^

^1^ Laboratoire d'Excellence « CORAIL », EPHE, PSL Research University, UPVD, CNRS, USR 3278 CRIOBE, F-66360 Perpignan, FRANCE

^2^ Instituto de Ciencias Ambientales y Evolutivas, Universidad Austral de Chile, Valdivia, Chile

^3^ Institut Systématique, Évolution, Biodiversité (ISYEB), UMR 7205, CNRS, MNHN, UPMC, EPHE, Ecole Pratique des Hautes Etudes, Sorbonne Universités, 57 rue Cuvier, CP39, F-75005, Paris, France.

^4^ EPHE, PSL Research University, Paris, France

^5^ SymbioSeas and Marine Applied Research Center, Wilmington NC 28411, United States of America

^*^Author for correspondence: Erwan Delrieu-Trottin. e-mail: erwan.delrieu.trottin@gmail.com

**Figure S1:** EBSP representing the median of the NeT through time in years for all type of marker (mitochondrial (purple), nuclear (green)). Line shape denotes species that display a significant expansion (plain) from species that display no significant change in their population size, *i.e.* constant population size (dotted).

**Figure S2:** Histogram representing for all endemic and widespread species expansion times retrieved for mitochondrial (mtDNA) and nuclear (nucDNA) markers.

**Figure S3:** EBSP representing the median of the NeT through time in years for all families and type of marker (mitochondrial (red), nuclear (blue)). Line shape denotes species that display a significant expansion (plain) from species that display no significant change in their population size, *i.e.* constant population size (dotted).

**Figure S1**

**FigureS2**

**Figure S3**
